# Needs of older persons living in long-term care institutions: on the usefulness of cluster approach

**DOI:** 10.1186/s12877-021-02259-x

**Published:** 2021-05-17

**Authors:** Sławomir Tobis, Krystyna Jaracz, Sylwia Kropińska, Dorota Talarska, Juanita Hoe, Katarzyna Wieczorowska-Tobis, Aleksandra Suwalska

**Affiliations:** 1 Department of Occupational Therapy, Poznan University of Medical Sciences, ul. Swiecickiego 6, 60-781 Poznan, Poland; 2 Chair of Nursing, Poznan University of Medical Sciences, ul. Smoluchowskiego 11, 60-179 Poznan, Poland; 3 Geriatrics Unit, Chair and Department of Palliative Medicine, Poznan University of Medical Sciences, Poznan, Poland; 4 Chair of Preventive Medicine, Poznan University of Medical Sciences, ul. Swiecickiego 6, 60-781 Poznan, Poland; 5 Division of Nursing, School of Health Sciences, City, University of London, Northampton Square, London, EC1V 0HB UK; 6 Department of Mental Health, Chair of Psychiatry, Poznan University of Medical Sciences, ul. Szpitalna 27/33, 60-572 Poznan, Poland

**Keywords:** Needs, Care homes, Long-term care, Clusters, CANE

## Abstract

**Background:**

Long-term care units’ residents do not constitute a homogeneous population. Providing effective care, tailored to individual needs, is crucial in this context. It can be facilitated by suitable tools and methods, which include needs assessment along with the physical, psychological and social aspects of care. We thus applied a cluster approach to identify their putative groupings to enable the provision of tailored care.

**Methods:**

The needs of 242 residents of care homes in four Polish cities (Poznan, Wroclaw, Bialystok and Lublin), aged 75–102 years (184 females), with the Mini-Mental State Examination (MMSE) score ≥ 15 points, were assessed with the CANE (Camberwell Assessment of Need for the Elderly) questionnaire. Their independence in activities of daily living was evaluated by the Barthel Index (BI), and symptoms of depression by the Geriatric Depression Scale (GDS). The results of MMSE, BI and GDS were selected as variables for K-means cluster analysis.

**Results:**

Cluster 1 (C1), *n* = 83, included subjects without dementia according to MMSE (23.7 ± 4.4), with no dependency (BI = 85.8 ± 14.4) and no symptoms of depression (GDS = 3.3 ± 2.0). All subjects of cluster 2 (C2), *n* = 87, had symptoms of depression (GDS = 8.9 ± 2.1), and their MMSE (21.0 ± 4.0) and BI (79.8 ± 15.1) were lower than those in C1 (*p* = 0.006 and *p* = 0.046, respectively). Subjects of cluster 3 (C3), *n* = 72, had the lowest MMSE (18.3 ± 3.1) and BI (30.6 ± 18,8, *p* < 0.001 vs. C1 & C2). Their GDS (7.6 ± 2.3) were higher than C1 (*p* < 0.001) but lower than C2 (*p* < 0.001). The number of met needs was higher in C2 than in C1 (10.0 ± 3.2 vs 8.2 ± 2.7, *p* < 0.001), and in C3 (12.1 ± 3.1) than in both C1 and C2 (*p* < 0.001). The number of unmet needs was higher in C3 than in C1 (1.2 ± 1.5 vs 0.7 ± 1.0, *p* = 0.015). There were also differences in the patterns of needs between the clusters.

**Conclusions:**

Clustering seems to be a promising approach for use in long-term care, allowing for more appropriate and optimized care delivery. External validation studies are necessary for generalized recommendations regarding care optimization in various regional perspectives.

## Background

At a certain point, many people, for various reasons, face the necessity to relocate to a care institution [1]. As long as they live in the community, they usually have a high level of engagement in occupational activities and purpose in life [2]. They are anxious about the prospect of moving and are afraid of being unable to carry on with their daily habits and routines [3]. A move to a care home is described as *the final frontier*, triggering negative and distressing thoughts and provoking strong feelings [4]. Modern and effective approaches to long-term care (LTC) that secure both the efficacy of care and the best possible comfort for the residents are subsequently required.

Once in an LTC institution, older people attempt to establish a home there. Four categories have been identified as critical to this process: *continuity, preserving personal identity, belonging,* and *being active and working* [5]. Maintaining these is not easy, as the institutional framework of LTC settings can limit and restrict older people’s functioning [6]. Lack of autonomy upholds the stereotype of residents as (passive) recipients of care [7]. Behuniak states that the lack of control provokes a sense of powerlessness, and those with dementia are especially vulnerable [8]. The majority of residents in LTC units have dementia [9], and many of them report having a poor quality of life [10].

Residents of LTC facilities are commonly perceived as a relatively homogeneous group (usually physically unwell, with multiple functional deficits) [4], which is in contrast to the contemporary concept of providing individualized, person-centered care [11, 12]. As literature shows, LTC residents, in fact, constitute a diversified population [13, 14]. Providing effective care, tailored to individual needs, is crucial in this context [15] and can be facilitated by suitable tools and methods, which include needs assessment along with the physical, psychological and social aspects of care.

The Camberwell Assessment of Need for the Elderly (CANE) questionnaire can provide a basis for such an approach [16]. It encompasses the definition of need as a remediable deficit and includes both health and socio-economic needs, which are essential for a holistic approach. An important advantage of CANE is its ability to distinguish between needs receiving adequate support (met needs) and those for which optimal interventions are missing (unmet needs). As far as LTC facilities are concerned, a number of studies using CANE have demonstrated that the total number of their residents’ needs is generally high [17–20].

While each care setting’s population presents a unique distribution of needs, there would be value in being able to categorize those needs (and, subsequently, the residents) within a few ranges (or groups), with the care provision in mind. We, therefore, applied a cluster approach to investigate whether distinct cognitive, functional and psychological profiles exist, which can distinguish the needs of residents. If so, this could create a starting point for assigning residents in respect of need-based groups. Such a distinction could be used to plan and improve the care provided and could enable precisely addressed interventions. The significance of implementing care models and services which are derived from the needs of older people has been recently stressed by Abdi et al. [21]

To the best of our knowledge, cluster analysis has never been used to characterize older people’s needs assessed with CANE.

## Methods

We applied a cluster approach to identify the putative groupings of LTC residents based on their scores in CANE and selected tools of Comprehensive Geriatric Assessment.

### The participants

A total of 400 older individuals aged ≥75 years, living in LTC institutions in four Polish cities (Poznan, Wroclaw, Bialystok and Lublin), were included in the study. The randomization and study protocol have been previously described in detail [20].

For the analysis, the data of 242 subjects with Mini-Mental State Examination (MMSE) score of at least 15 (adjusted for age and education), which was considered sufficient to understand the questions asked and give reliable answers [22], were included. The data comprised age and sex of the respondents, their education level and length of stay in the institution, as well as the scores of geriatric assessment tools (Barthel Index – BI, Geriatric Depression Scale – GDS, MMSE, and the results of the CANE questionnaire).

### The assessment tools

BI was used for the assessment of dependence in basic activities of daily living [23]; the value of 80 points or more (out of the maximum of 100) was referred to as no dependence [24].

The short version of GDS (15 items) served as the screening tool for depression [25]. Subjects with at least 6 points (out of the maximum of 15) were classified as having symptoms of depression [26].

MMSE, a brief screening assessment tool, was used for detecting subjects with increased risk of dementia [27]. Possible scores range from 0 to 30: 27–30 being considered normal, 24–26 meaning mild cognitive impairment without dementia, 20–23 – mild dementia, and 15–19 – moderate dementia. Obtained scores were adjusted for age and education [28].

The CANE questionnaire is a comprehensive tool developed for the assessment of needs in older adults. Structured interviews were performed face-to-face by researchers trained using the CANE manual [16]. We used the Polish version of the questionnaire, which had been proven to have good psychometric properties in a pilot study [29].

The questionnaire covers a total of 24 areas of social, medical, psychological, and environmental needs, as well as two additional domains regarding caregivers (which were not analyzed in this article). Each area poses a question about a particular need. Responses are rated on a three-point scale where 0 means no need, 1 – met need (i.e., the problem is receiving or has received a proper intervention), and 2 – unmet need (i.e., the problem is left without appropriate intervention). For each participant, the numbers of met and unmet needs, as well as the number of all needs (sum of met and unmet needs), were calculated.

### Statistical analysis

The statistical analysis was performed with STATISTICA 13.0 (StatSoft, Poland) and SPSS software (IBM, Poland). For all characteristics analyzed, means and standard deviations were calculated. Normality in the data distribution was examined with the Shapiro-Wilk’s test. Due to the lack of normality, medians were also presented. *P* < 0.05 was considered to indicate statistical significance, and *p* between 0.05 and 0.10 – an insignificant trend.

### K-means cluster analysis

K-means clustering is a way to use data to uncover natural groupings within a heterogeneous population. To reveal the patterns, the algorithm starts by first assigning data points into random groups. The group centers are then calculated, and the group memberships are reassigned based on the distances between each data point and the group centers. This process is repeated until there are no changes in the group memberships from the previous iteration.

The results of the MMSE, BI and GDS were selected as variables for cluster analysis. K-means clustering was chosen as the clustering method since it is uniquely designed for non-hierarchical data partitioning (such as our data); in our case, it generated a small number of subgroups in studied LTC residents. As the abovementioned variables have been measured on different scales with different score ranges, all scores were transformed into z-scores before introducing them to the cluster analysis. Differences between cluster profiles were identified using χ^2^ for categorical variables and analyses of variance (F-tests) for continuous variables.

## Results

The mean age of studied subjects was 83.1 ± 5.9 years (median: 83.0 years; range: 75–102 years); 184 of them were females (76.0%). The mean length of institutionalization was 73.2 ± 66.7 months (median: 60.0 months; range: 1–299 months). The mean BI was 67.2 ± 29.0 points (median: 75.0 points; range: 0–100 points), MMSE – 21.4 ± 4.5 points (median: 21.0 points; range: 15–30 points), and GDS – 6.6 ± 3.2 points (median: 7.0 points, range: 0–14 points). Detailed characteristics of the studied group are presented in Table 1.

**Table 1:** The characteristics of studied subjects

Parameter	Characteristic	n (%)
Age	75–79 years	75 (31.0)
	80–84 years	80 (33.0)
	85–89 years	58 (24.0)
	90+	29 (12.0)
Education	Primary	112 (46.3)
	Secondary	96 (39.7)
	Higher (at least bachelor degree)	24 (9.9)
	Lack of data	10 (4.1)
Length of institutionalization	Less than one year	34 (14.0)
	Between 1 and 5 years	88 (36.4)
	Between 5 and 10 years	63 (26.0)
	More than ten years	53 (21.9)
	Lack of data	4 (1.7)
MMSE	15–23 points	161 (66.5)
	24–30 points	81 (33.5)
BI	0–20 points	24 (9.9)
	21–80 points	129 (53.3)
	Above 80 points	89 (36.8)
GDS	0–5 points	86 (35.5)
	6–10 points	126 (52.1)
	Above 10 points	30 (12.4)

Note. *MMSE* Mini Mental State Examination, *BI* Barthel Index, *GDS* Geriatric Depression Scale

### Analysis of needs

The mean number of needs in all studied subjects was 10.9 ± 3.2. Of these, a mean 10.0 ± 3.1 were met and 0.9 ± 1.2 – unmet needs. Met needs were observed for more than 90% of studied subjects in the following areas *Looking after the home* (*n* = 235–97.1%)*, Food* (*n* = 234–96.7%) and *Physical health* (*n* = 227–93.8%). Met needs were also commonly noted (i.e., ≥50% of studied subjects) for: *Accommodation* (*n* = 217–89.7%), *Self-care* (*n* = 179–74.8%), *Money/budgeting* (*n* = 154–63.6%), *Mobility/falls* and *Continence* (both *n* = 153–63.2%), and *Eyesight/hearing/communication* (*n* = 141–53.8%). Unmet needs were most commonly reported for: *Psychological distress* (*n* = 42–17.4%), *Company* (*n* = 41–16.9%), *Eyesight/hearing/communication* and *Intimate relationship* (both *n* = 29–12.0%), and *Daytime activities* (*n* = 28–11.6%). In the remaining areas, unmet needs were recognized in no more than 10% of studied subjects.

### Cluster analysis

Three clusters were identified based on concurrent analysis of the MMSE, BI and GDS scores.

**Cluster 1** subjects (*n* = 83) had a mean MMSE score of 23.7 ± 4.4 points (i.e., above the cut-off point for dementia), BI score – 85.8 ± 14.4 (i.e., no dependency) and GDS score – 3.3 ± 2.0 (i.e., no symptoms of depression). In this cluster, 33 subjects (39.8%) had MMSE scores within the normal range, and only 19 (22.9%) scored below 20 points. Thirty-one individuals (37.3%) had the maximum score in BI (100 points), whereas the minimum score of this group was 45 points. Moreover, only six subjects (7.2%) of this cluster had symptoms of depression.

**Cluster 2** subjects (*n* = 87) all had symptoms of depression; the mean GDS was 8.9 ± 2.1 points (*p* < 0.001 vs cluster 1). They had lower mean MMSE (21.0 ± 4.0 points – *p* = 0.006) and lower mean BI (79.8 ± 15.1 points – *p* = 0.046) compared to those of cluster 1. Only 20 subjects (23.0%) had MMSE scores in the normal range, and 25 (28.7%) had these results below 20 points. For the BI, 13 participants (14.9%) achieved the maximum score, and the minimum score was 40.

**Cluster 3** subjects (*n* = 72) had mean MMSE scores of 18.3 ± 3.1 (*p* < 0.001 vs. cluster 1 & 2), mean BI – 30.6 ± 18.8 points (*p* < 0.001 vs. cluster 1 & 2) and mean GDS – 7.6 ± 2.3 (*p* < 0.001 vs. cluster 1 & 2). Only one participant in that group had their MMSE result within the normal range. Most subjects of cluster 3 had MMSE scores below 20 points (*n* = 50, 69.4%). The maximum BI of that cluster was 65 points, and 61 subjects (84.7%) had symptoms of depression (GDS score ≥ 6 points).

For the length of institutionalization, no significant difference was observed between the clusters (cluster 1: mean 73.2 ± 65.2 months, cluster 2: mean 76.2 ± 67.9 months, cluster 3: mean 69.1 ± 66.7 months). However, subjects in cluster 3 were slightly older in comparison with those of cluster 1 (mean 84.4 ± 6.5 years vs 82.0 ± 5.8 years; *p* = 0.049); the age difference vs cluster 2 (mean 82.5 ± 5.9 years) was not significant.

### Met and unmet needs in the clusters

Each cluster had a distinct pattern of needs. The number of needs differed significantly between the clusters. The total number of needs was lowest in cluster 1 and highest in cluster 3 (*p* < 0.001). Similar results were observed for met needs (*p* < 0.001). The number of unmet needs differed significantly between clusters 1 and 3 (*p* = 0.015); between clusters 2 and 3, only a trend was observed (*p* = 0.056). Detailed characteristics of needs are presented in Table 2.

**Table 2:** The characteristics of needs in analyzed clusters

	Number of met needs means ± SD	Number of unmet needs means ± SD	Number of all needs means ± SD
**Cluster 1**	8.2 ± 2.7	0.7 ± 1.0	8.9 ± 2.7
**Cluster 2**	10.0 ± 3.2 *p < 0.001* vs. *cluster 1*	0.8 ± 1.1	10.8 ± 3.1 *p < 0.001* vs. *cluster 1*
**Cluster 3**	12.1 ± 3.1 *p < 0.001* vs. *cluster 1 & 2*	1.2 ± 1.5 *p = 0.015* vs. *cluster 1* *p = 0.056* vs. *cluster 2*	13.3 ± 2.0 *p < 0.001* vs. *cluster 1 & 2*

Characteristics of met needs within the clusters are presented in Table 3. Clusters 1 and 2 differed significantly in four areas, whereas clusters 1 and 3 – in as many as 11 areas.

**Table 3:** Detailed characteristics of met needs in analyzed clusters

Area		Cluster 1 ***n*** = 83	Cluster 2 ***n*** = 87	Cluster 3 ***n*** = 72	
**1**	**Accommodation**	73 (88%)	75 (86%)	69 (96%)	*NS*
**2**	**Looking after the home**	77 (93%)	86 (99%)	72 (100%)	*1* vs. *3 p = 0.031*
**3**	**Food**	79 (95%)	84 (97%)	71 (99%)	*NS*
**4**	**Self-care**	46 (55%)	61 (70%)	72 (100%)	*1* vs. *3 p < 0.001* *2* vs. *3 p < 0.001*
**5**	**Caring for someone else**	1 (1%)	2 (2%)	1 (1%)	*NS*
**6**	**Daytime activities**	27 (33%)	43 (49%)	48 (67%)	*1* vs. *2 p = 0.030* *1* vs. *3 p < 0.001* *2* vs. *3 p = 0.036*
**7**	**Memory**	24 (29%)	37 (43%)	50 (69%)	*1* vs. *3 p < 0.001* *2* vs. *3 p < 0.001*
**8**	**Eyesight/hearing/communication**	37 (45%)	52 (60%)	52 (72%)	*1* vs. *3 p < 0.001*
**9**	**Mobility/falls**	37 (45%)	53 (61%)	63 (88%)	*1* vs. *2 p = 0.045* *1* vs. *3 p < 0.001* *2* vs. *3 p < 0.001*
**10**	**Continence**	33 (40%)	51 (59%)	69 (96%)	*1* vs. *2 p = 0.015* *1* vs. *3 p < 0.001* *2* vs. *3 p < 0.001*
**11**	**Physical health**	77 (93%)	82 (94%)	68 (94%)	*NS*
**12**	**Drugs**	18 (22%)	25 (29%)	30 (42%)	*1* vs. *3 p = 0.009*
**13**	**Psychotic symptoms**	11 (13%)	17 (20%)	14 (19%)	*NS*
**14**	**Psychological distress**	26 (31%)	33 (38%)	31 (43%)	*NS*
**15**	**Information**	16 (19%)	31 (36%)	28 (39%)	*1* vs. *2 p = 0.025* *1* vs. *3 p = 0.008*
**16**	**Deliberate self-harm**	3 (4%)	10 (11%)	10 (14%)	*1* vs. *3 p = 0.038*
**17**	**Inadvertent self-harm**	2 (2%)	5 (6%)	3 (4%)	*NS*
**18**	**Abuse/neglect**	3 (4%)	1 (1%)	1 (1%)	*NS*
**19**	**Behavior**	6 (7%)	10 (11%)	8 (11%)	*NS*
**20**	**Alcohol**	3 (4%)	3 (3%)	2 (3%)	*NS*
**21**	**Company**	23 (28%)	26 (30%)	31 (43%)	*NS*
**22**	**Intimate relationship**	3 (4%)	3 (3%)	1 (1%)	*NS*
**23**	**Money/budgeting**	40 (48%)	55 (63%)	59 (82%)	*1* vs. *3 p = 0.002*
**24**	**Benefits**	18 (22%)	26 (30%)	15 (21%)	*NS*

The comparison of unmet needs in analyzed clusters is shown in Table 4 (only for the areas in which ≥10% of subjects had unmet needs). In the area *Daytime activities,* the subjects of cluster 3 had more unmet needs in comparison with both cluster 1 and 2 (*p* = 0.023 and *p* = 0.003, respectively). There were also differences in the areas *Eyesight/hearing/communication* (cluster 2 vs. cluster 3, *p* = 0.022) and *Psychological distress* (cluster 1 vs. cluster 3, *p* = 0.032).

**Table 4:** Detailed characteristics of unmet needs in analyzed clusters (the areas in which more than 10% of subjects had unmet needs)

Area		Cluster 1 ***n*** = 83	Cluster 2 ***n*** = 87	Cluster 3 ***n*** = 72	
**6**	**Daytime activities**	5 (6%)	6 (7%)	17 (24%)	*2* vs. *3 p = 0.023* *1* vs. *3 p = 0.003*
**8**	**Eyesight/hearing/communication**	11 (13%)	5 (6%)	13 (18%)	*2* vs. *3 p = 0.022*
**14**	**Psychological distress**	9 (11%)	15 (17%)	18 (25%)	*1* vs. *3 p = 0.032*
**21**	**Company**	9 (11%)	19 (22%)	13 (18%)	*NS*
**22**	**Intimate relationship**	9 (11%)	7 (8%)	13 (18%)	*NS*

## Discussion

The usefulness of CANE for the assessment of needs in residents of LTC institutions has been demonstrated previously [16, 18, 19]. The overall number of needs in these individuals was reported to be higher than in those living in the community [30, 31]. These observations are in agreement with our results showing that the mean number of needs reached almost eleven.

We found that, despite a high number of total needs, only a few were unmet, which suggests good quality care was being provided. Van der Ploeg et al. [32] likewise suggested that low numbers of unmet needs were due to the appropriateness of care provision. The unmet needs we observed had similar patterns of distribution to those reported previously in nursing homes [19].

In our study, LTC residents were grouped using cluster analysis based on their cognitive, functional and psychological status (assessed with MMSE, BI, and GDS) with regard to their needs (assessed with CANE). As the scores used for the assessment of the functional and psychological status of a person do not change proportionally to each other [19, 33], it is right that they are included as independent factors in the analysis. To the best of our knowledge, such an analysis has not been done before. Each of the recognized clusters identified a group of subjects with a distinct profile of physical dependency, cognitive status, symptoms of depression, and pattern of needs. In the care practice, the distinction of such groups of residents allows for accurate allocation of human and material resources to match the residents’ needs at both individual and group levels.

Cluster 1 subjects had the lowest number of needs, were functionally independent and cognitively intact, with few depressive symptoms. Still, they had almost nine needs, and most of these were met. The number of needs in this cluster reflects the factors leading to placement in an LTC institution [34]. Residents within this group should be evaluated periodically to assess if they really need to stay in LTC units or should rather be provided with intermediate care of appropriate intensity. The development of alternative intermediate care settings, which allow flexibility of the degree of care, is recommended [34].

The presence of depressive symptoms was the common denominator of our cluster 2 subjects. The group in this cluster had more met needs than those in cluster 1, which indicates higher use of care resources. The residents of LTC settings often have difficulty finding purpose in life, which can lead to the development of depressive symptoms and a declining ability to perform activities of daily living [35]. The distinction of this cluster signals the importance of recognizing and treating depression, as it impacts both independence [36] and needs [19]. Certainly, some of these subjects may require pharmacological treatment of their depression, but a significant number can be helped through non-pharmacological interventions, which may restore meaningfulness in their lives. According to the evidence, non-pharmacological interventions had positive effects on depressive [37] and cognitive [38] symptoms in older adults. Providing appropriate interventions means some residents could potentially move category from cluster 2 to cluster 1 (thereby reducing their care requirements).

Cluster 3, which constitutes the core group of LTC clients, consists of subjects with higher cognitive and physical impairments. They had more needs, both met and unmet than those in the other two clusters. This is consistent with a previous study, which found that the number of needs correlated with functional impairment [17]. A strong negative correlation between the sum of met and unmet needs and the BI has also previously been found [33]. Individuals in our cluster 3 had significantly more unmet needs in the area *Daytime activities* than those in clusters 1 and 2. This indicates the need to include residents in increased activities tailored to the individuals’ abilities [39] and enable the residents to participate actively [40]. Examples comprise involving residents in everyday chores, which are commonly associated with normality [41]. Such an approach sensitizes the staff to view the resident as a whole person, not just a set of limitations to be cared for [42]. The mental well-being of subjects with dementia is significant, as lower mood rather than the level of dependency has a greater influence on residents’ quality of life [43], and dementia also appears to be one of the most important factors contributing to functional decline in nursing home residents [44].

The limitations of our study result from the fact that we analyzed individuals who were cognitively well functioning alongside those with symptoms of moderate dementia and excluded those with symptoms of severe and moderately severe dementia. The needs of residents with more advanced dementia are complex and may include palliative care needs, which should be investigated separately. The cross-sectional design of our study additionally means that the results may point toward important relationships but cannot imply causality. Also, the generalizability of findings may be limited due to the sample size and the variability of the prevalence of severe dementia in LTC facilities between countries. However, the strength of the study is the inclusion of “older old” subjects, aged 75 years or more, who constitute a more demanding subgroup than the “younger old” as far as care delivery in LTC institutions is concerned, and who are often underrepresented in studies with older adults.

## Conclusion

Our study presents a novel approach to the assessment and categorization of LTC residents. The method of cluster analysis was effective in identifying groups of residents for whom intervention was possible to improve their functioning and subsequently decrease the number of needs. It provides a better understanding of complex relationships between the functional and psychological status and the needs of the residents. The insights gained allow for better focused, tailored care delivery and more appropriate, personalized interventions. This method could also be useful for individuals newly admitted to LTC settings, enabling them to be assigned the appropriate care package.

The gains from our work are thus two-fold: scientific (moving forward the understanding of the structure of LTC units’ residents, and inspiring further investigations in this matter), and practical (the persons in charge of LTC acquire valuable information regarding the residents, especially in the context of identification of clusters for which targeted and optimized care can be provided). Yet, for generalized recommendations regarding care optimization in various regional perspectives, external validation studies are necessary.

## Data Availability

All datasets used and analyzed during the current study are property of Poznan University of Medical Sciences, Poznan, Poland. The database is available from the corresponding author on reasonable request.

## Abbreviations

BI: Barthel Index
CANE: Camberwell Assessment of Need for the Elderly
GDS: Geriatric Depression Scale
LTC: Long term care
MMSE: Mini-Mental State Examination
